# Hearing in the Juvenile Green Sea Turtle (*Chelonia mydas*): A Comparison of Underwater and Aerial Hearing Using Auditory Evoked Potentials

**DOI:** 10.1371/journal.pone.0159711

**Published:** 2016-10-14

**Authors:** Wendy E. D. Piniak, David A. Mann, Craig A. Harms, T. Todd Jones, Scott A. Eckert

**Affiliations:** 1 Division of Marine Science and Conservation, Duke University Marine Lab, Beaufort, North Carolina, United States of America; 2 College of Marine Science, University of South Florida, St. Petersburg, Florida, United States of America; 3 Center for Marine Sciences and Technology, College of Veterinary Medicine, North Carolina State University, Morehead City, North Carolina, United States of America; 4 Department of Zoology, University of British Columbia, Vancouver, British Columbia, Canada; 5 Wider Caribbean Sea Turtle Conservation Network, Ballwin, Missouri, United States of America; 6 Department of Biology and Natural Resources, Principia College, Elsah, Illinois, United States of America; Kyoto University, JAPAN

## Abstract

Sea turtles spend much of their life in aquatic environments, but critical portions of their life cycle, such as nesting and hatching, occur in terrestrial environments, suggesting that it may be important for them to detect sounds in both air and water. In this study we compared underwater and aerial hearing sensitivities in five juvenile green sea turtles (*Chelonia mydas*) by measuring auditory evoked potential responses to tone pip stimuli. Green sea turtles detected acoustic stimuli in both media, responding to underwater stimuli between 50 and 1600 Hz and aerial stimuli between 50 and 800 Hz, with maximum sensitivity between 200 and 400 Hz underwater and 300 and 400 Hz in air. When underwater and aerial hearing sensitivities were compared in terms of pressure, green sea turtle aerial sound pressure thresholds were lower than underwater thresholds, however they detected a wider range of frequencies underwater. When thresholds were compared in terms of sound intensity, green sea turtle sound intensity level thresholds were 2–39 dB lower underwater particularly at frequencies below 400 Hz. Acoustic stimuli may provide important environmental cues for sea turtles. Further research is needed to determine how sea turtles behaviorally and physiologically respond to sounds in their environment.

## Introduction

While the biological significance of hearing in sea turtles remains largely unstudied, sea turtles are able to detect [1–5] and respond to acoustic stimuli [4–8], and may use sound for navigation, locating prey, avoiding predators, and general environmental awareness. Sea turtles spend much of their life underwater, but they breathe at the air-water interface and critical portions of their reproductive cycle (egg laying and hatching) take place on land. Thus, it may be important that sea turtles be able to detect sound in both underwater and aerial environments.

Sea turtles lack external pinnae or ear canals and, like other Testudines, their ear is covered by an extension of facial tissue called the tympanum. Unlike terrestrial turtles and tortoises marine turtles have a thick layer of subtympanal fat and connective tissue [9]. The middle ear is surrounded by bone, filled with air, and connected to the throat via the Eustachian tube. The sea turtle ossicular mechanism is comprised of the extracolumella and the columella (stapes). The mushroom-shaped, cartilaginous extracolumella lies beneath the tympanum and is connected by ligaments to the columella. The columella (stapes) is a long, thin, curved bone, encased in a narrow, bony channel, which extends medially into the middle ear, through the fluid-filled pericapsular recess to the oval window, where it expands to form a large, cone-shaped footplate [9]. Small, fibrous stapedosaccular strands, which are unique to turtles and hypothesized to relay vibrational energy, connect the stapes and oval window to the saccule [9–11]. Inward and outward movement of the columella causes movement of fluid in the pericapsular recess, stimulating hair cells located on the basilar membrane and limbus of the cochlea [9].

The functional morphology of the sea turtle ear remains poorly understood and despite previous anatomical research it is still unclear whether sea turtle ears respond to pressure, particle motion, or both. Observational studies of the thick tympanum in sea turtles found little tympanic displacement in response to sound, and concluded that turtle ears had little capacity for impedance matching in air [11]. Computerized tomography of sea turtle subtympanal fat revealed that it is similar to the fat found in the middle ears of marine mammals and birds. The density of these fats is consistent with sound speeds in seawater, suggesting the subtympanal fat layer may act as a low-impendence channel for conduction of underwater sound to the middle and inner ears [12]. Lenhardt et al. [10, 13] proposed that the sea turtle ear is adapted for hearing via bone conduction in water and is a poor receptor in air, suggesting that the whole body serves as a receptor while the turtle is underwater. However, evidence derived from research on freshwater turtles suggests a more typical tympanic middle ear pathway for sound in sea turtles [14]. Research on freshwater aquatic turtles has shown that aerial and vibrational stimuli produce different audiograms and that turtles are more sensitive to aerial, rather than vibrational stimuli [15–17]. Removal or cutting of the columella drastically reduced aerial hearing sensitivity, but only slightly reduced vibrational hearing sensitivity [16]. Both auditory and vibrational stimuli appeared to be processed by the auditory system, likely combining to create a single electrophysiological response [15].

Electrophysiological and behavioral studies have demonstrated that sea turtles are able to detect low-frequency acoustic stimuli. Ridgway et al. [1] collected the first measurements of sea turtle hearing sensitivity by recording cochlear response potentials to aerial and vibrational stimuli in three juvenile green turtles (*Chelonia mydas*). Turtles responded to aerial stimuli between 50 and 2000 Hz and vibrational stimuli between 30 and 700 Hz, with maximum sensitivity between 300 and 500 Hz for both stimuli. Ridgway et al. [1] suggested that the “useful” frequency span of the green turtle ear was between 60 and 1000 Hz. More recent measurements of sea turtle hearing sensitivity have been made by recording auditory evoked potentials (AEPs). AEPs are an electrical response produced by the central auditory nervous system after stimulation by sound detectable by the ear [18–19]. Bartol et al. [2] measured short latency AEPs (auditory brainstem responses) in juvenile loggerhead sea turtles (*Caretta caretta*) in response to two types of vibrational stimuli: low-frequency clicks and tone bursts delivered directly to the tympanum. They measured a mean click threshold of -10.8 dB re: 1g rms ± 2.3 dB SD and an effective hearing range from tone bursts from 250 to 750 Hz with maximum sensitivity at 250 Hz, the lowest frequency tested [2]. AEP measurements of hearing sensitivity in partially submerged sea turtles in response to aerial stimuli found Pacific sub-adult green turtles responded to stimuli between 100 and 500 Hz, with highest sensitivity between 200 and 400 Hz [3]. In the same study, Atlantic juvenile green turtles responded to stimuli between 100 and 800 Hz, with highest sensitivity between 600 and 700 Hz, and juvenile Kemp’s ridley sea turtles (*Lepidochelys kempii*) responded to stimuli between 100 and 500 Hz with maximum sensitivity between 100 and 200 Hz [3]. In a comparative study examining the differences between AEP (electrophysiological) and behavioral response techniques for assessing hearing sensitivity, post-hatching and juvenile loggerhead sea turtles responded to underwater stimuli between 50 and 1100 Hz, with lower thresholds detected using behavioral techniques [4]. In a similar study a single adult loggerhead sea turtle responded to underwater stimuli between 50 and 800 Hz with best sensitivity at 100 Hz using behavioral response techniques, and between 100 and 1131 Hz with best sensitivity between 200 and 400 Hz using AEP techniques [5].

Marine turtle ears are hypothesized to be adapted for underwater sound detection, however few studies have focused on measuring underwater hearing sensitivity, or on comparing underwater and aerial hearing sensitivity. In an effort to increase our understanding of the amphibious hearing capabilities of sea turtles, in the present study we measured and compared the underwater and aerial hearing sensitivity of juvenile green sea turtles.

## Materials and Methods

### Sea Turtles

We measured the hearing thresholds of five Atlantic juvenile green turtles underwater and in air, by recording auditory evoked potentials (AEPs) at the Zoology Animal Care Center at the University of British Columbia in Vancouver, BC, Canada. Turtles averaged 34 kg in weight (range: 26–38 kg), 65 cm in curved carapace length (range: 60–67 cm) and 56.5 cm in curved carapace width (range: 53–60 cm). Each turtle was individually identified by markings on outer carapace scutes. For example, L3 corresponds to a turtle identified by a marking on the third scute on the left side of the carapace.

### Auditory evoked potential measurements

#### Underwater experimental setup

To prevent muscle movement that would mask AEPs, we lightly restrained the turtles by encasing them in a cloth bag before testing. Their heads were left exposed so the turtles could breathe normally. We completely submerged turtles to a depth of at least 10 cm (measured at the location of the ears) below the surface in a cylindrical fiberglass tank (2 m in diameter and 1.5 m in depth). We submerged an amplified speaker (AQ339 Aquasonic Underwater Speaker, Clark Synthesis, Inc., Littleton, Colorado USA; amplifier: Hafler P1000, Rockford Corporation, Tempe, Arizona USA) 40 cm away from and level with the turtle’s ears. During data collection, water temperatures were approximately 22°C.

#### Aerial experimental setup

We isolated turtles from noise and vibrations, lightly restrained them using a cloth bag to prevent muscle movement, and placed them on an angled resting board with their head free. We suspended an amplified speaker (AQ339 Aquasonic Underwater Speaker, Clark Synthesis, Inc.; amplifier: Hafler P1000, Rockford Corporation) 80 cm directly in front of the turtle and level with the turtle’s ears. To reduce the possibility of a vibratory response during in the air trials, the speaker was suspended on an elastic cord and positioned on a separate table. During data collection, air temperatures were approximately 21°C.

#### Auditory Evoked Potential Measurements with Anesthesia

We collected underwater AEPs on two turtles using anesthesia, and for one turtle we collected underwater AEPs with and without anesthesia. Anesthesia was induced with medetomidine 50 μg/kg and ketamine 5 mg/kg combined and injected intravenously into the dorsal cervical sinus. Turtles were intubated with a specially designed double-cuffed endotracheal tube with both proximal and distal cuffs forming a watertight seal and preventing the cuff from slipping in the trachea. We ventilated the turtles manually with a 1.5 L ambu bag at a rate of two breaths in quick succession every two to three minutes, with additional ventilations during gaps in the AEP collection. Ventilation rate and volume were based on observations of voluntary respirations of manually restrained turtles [20], and from reported respiratory rates and tidal volumes (39 ml/kg) of green sea turtles [21]. At the completion of AEP measurements we reversed the anesthesia with atipamezole 0.25 mg/kg half IV and half IM (see Harms et al. [20] for further anesthesia details). To evaluate the efficacy of using anesthesia as a restraint for the collection of AEPs, we compared resulting audiograms and venous blood gas values before and after the procedures for anesthetized and unanesthetized turtles.

#### Signal generation and recording of auditory evoked potentials

To record AEP signals, we inserted needle electrodes (27 ga, 12 mm in length, Rochester Electro-Medical, Inc., Lutz, Florida USA) subdermally on the top of the head under the frontal scale (recording electrode); in the deltoid muscle of the neck (reference electrode); and either in the deltoid muscle of the shoulder (air: ground electrode) or seawater (water: ground electrode). We used an Evoked Potential Workstation (Tucker-Davis Technologies, Inc. Alachua, Florida USA) and laptop computer with SigGenRP and BioSigRP software (Tucker-Davis Technologies, Inc.) to generate tonal stimuli, and recorded AEP responses from the electrodes at a sampling rate of 24412 Hz. We amplified signals from the electrodes using a digital biological amplifier (Tucker-Davis Technologies, Inc.) and filtered the signals to remove sound outside the frequencies of interest (high pass: 50 Hz; low pass: 5 kHz; band reject: 60 Hz). Electrode impedances were less than 3 kΩ. We presented pulsed sinusoidal tonal stimuli, 50 ms in length, shaped with a Hanning window, at a rate of 13 s^-1^, with alternating phase. We recorded responses to frequencies between 50 and 3200 Hz, and attenuated stimuli in 6 dB steps beginning at the highest level that could be generated at each frequency and attenuating until no further AEP signal could be identified (after up to 1000 AEP signal averages). To increase the number of recordings for each individual, we advanced to the next reduced sound pressure level if an AEP response was detected before 1000 signal averages. We paused recordings when the turtles moved or lifted their heads to breathe to ensure we made all measurements with the turtle’s head in the same position in the acoustic field.

#### Calibration

We calibrated the sound field using a hydrophone (HTI-96-min, High Tech, Inc. Gulfport, Mississippi USA; sensitivity: -164 dBV/μPa; 0.02–30 kHz) placed at the location of the turtle's head, but with the turtle absent. The hydrophone response in air was later calibrated against a sound level meter (330–2050, RadioShack, Fort Worth, Texas USA) (hydrophone aerial sensitivity: -126 dBV/20 μPa: 50–3200 Hz). Calibrations were made using two RP2.1 modules and BioSigRP (Tucker-Davis Technologies, Inc.) which repeatedly played the signal at the same rate used while recording AEPs, and simultaneously recorded the hydrophone signal at sampling rate of 24414 Hz. In water this procedure accounts for reverberation in the tank, as opposed to calibrating with long duration tones. We measured the background noise level using FieldLog (custom software, David Mann, University of South Florida) at a sampling rate of 24414 Hz using the RP2.1 with the HTI-96-min, and analyzed background noise frequency spectra using MATLAB (version 7.14, MathWorks, Inc. Natick, Massachusetts 01760 USA).

### Data Analyses

Because the presence of low-frequency background and electrical noise caused inaccuracies in automated threshold detection, we performed threshold analyses manually, a method commonly used in hearing investigations using AEPs (e.g. [4–5, 22–24]). We used BioSipRP (Tucker- Davis Technologies, Inc.) and Matlab software (MathWorks, Inc.) to make visual inspections of AEP responses in the time domain and analyzed the presence or absence of AEP signals using 2048-point fast Fourier transforms (FFTs) in the frequency domain. An AEP was determined to be present if the signal showed a peak twice that of the stimulus frequency (e.g. a peak at 600 Hz when the stimulus presented was 300 Hz) at least 6 dB above the noise floor 100 Hz on either side of peak in the frequency domain. We defined threshold as the lowest sound level at which a peak in the FFT was recorded.

### Ethics Statement

The green sea turtles were imported from the Cayman Turtle Farm (1983; Grand Cayman, British Virgin Islands; CITES Export Permit 2002/ky/000112) to the Zoology Animal Care Center, Department of Zoology, University of British Columbia (CITES Import Permit CA 02CWIM0129). This study was approved by the Institutional Animal Care and Use Committees of Duke University (Protocol #A235-07-08) and the University of British Columbia (Protocol #A07-0375).

## Results

### Auditory evoked potential waveform characteristics

**Fig 1:**
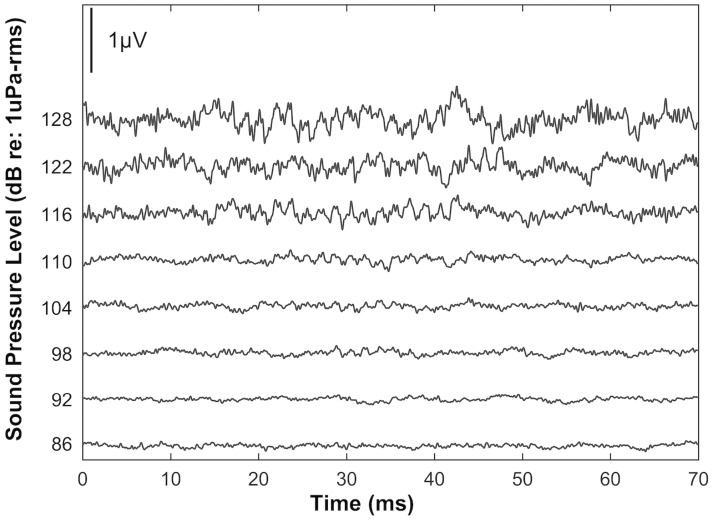
Underwater auditory evoked potential waveforms recorded from a juvenile green sea turtle (*Chelonia mydas*, L4), and corresponding stimuli levels in response to an underwater signal of 300 Hz.

**Fig 2:**
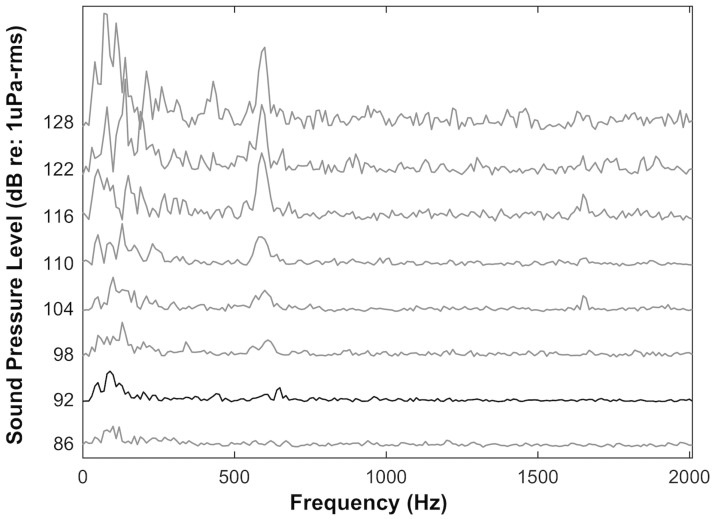
2048-point fast Fourier transforms of recorded auditory evoked potentials (presented in Fig 1) showing a peak at twice the frequency presented (600 Hz). Threshold level is presented in black (92 dB re: 1 μPa-rms).

**Fig 3:**
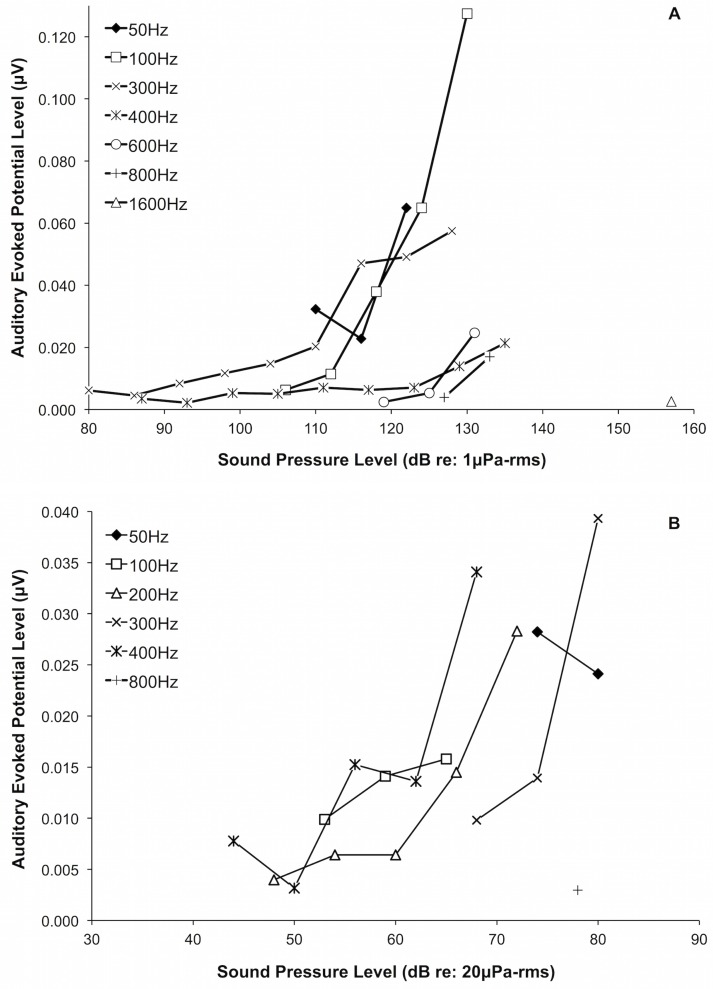
Juvenile green sea turtle (*Chelonia mydas*, L4) underwater (A) and aerial (B) input-output functions of auditory evoked potential level (μV) as a function of stimulus sound pressure level.

The AEP waveforms recorded from averaged responses to pulsed tonal signals in both air and water increased in latency and decreased in amplitude as we attenuated the stimuli (Fig 1). Recorded AEP waveforms were twice the frequency of the presented tonal stimuli (Fig 2), and AEP levels (μV) generally decreased with decreasing sound pressure levels in both air and water (Fig 3).

### Underwater audiograms

**Fig 4:**
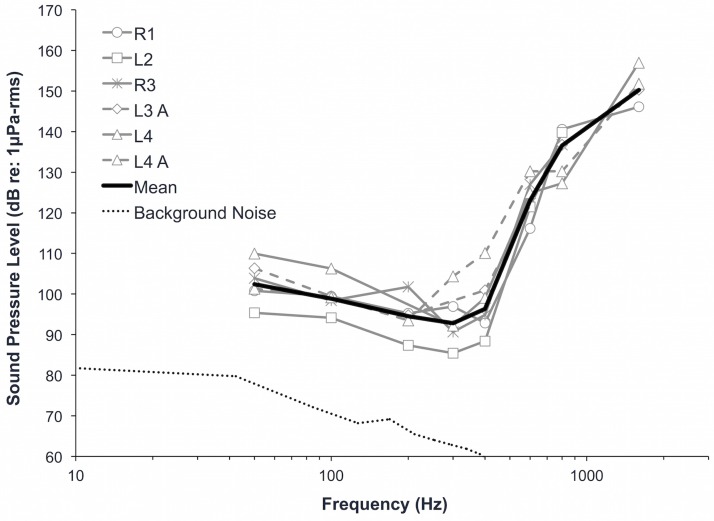
Underwater audiograms for juvenile green sea turtles (*Chelonia mydas*) in terms of pressure. “A” denotes the use of anesthesia when recording auditory evoked potentials. The hearing sensitivity of L4 was measured twice, with and without anesthesia (mean calculation uses the mean of these two measurements). Spectrum level background noise is represented by the dotted line (dB re: 1 μPa/√Hz).

Juvenile green turtles responded to signals between 50 and 1,600 Hz in water, with maximum sensitivity between 200 and 400 Hz (Table 1, Fig 4); sensitivity decreased sharply above 400 Hz. The lowest pressure sensitivity recorded was 85 dB re: 1 μPa-rms at 300 Hz (turtle L2). We found variation among individual turtles in terms of threshold levels as well as highest frequencies detected. Pressure threshold level differences among individuals varied between <1 and 19 dB, however up to 6 dB of this variability could be due to the step size used during the AEP measurements. All turtles responded to frequencies between 50 and 800 Hz, but only three responded to 1600 Hz. Background noise levels were <75 dB re: 1 μPa at 50 Hz, <63 dB re: 1 μPa at 300 Hz, and decreased as frequency increased.

**Table 1:** Underwater pressure thresholds (dB re: 1 μPa-rms) for individual juvenile green sea turtles (*Chelonia mydas*), and mean thresholds for all turtles combined. Frequencies tested with no detected auditory evoked potential response are presented with > “highest sound pressure level presented” (dB re: 1 μPa-rms).

Turtle	Frequency (Hz)								
ID	50	100	200	300	400	600	800	1600	3200
R1	101	99	95	97	93	116	141	146	-
L2	95	94	87	85	88	121	140	>150	>146
R3	104	98	102	91	95	127	137	>155	>147
L3 A	106	99	95	-	101	-	137	150	>151
L4	110	106	-	92	99	125	127	157	-
L4 A	101	99	93	104	110	130	130	152	>152
Mean^a^	102	99	95	93	96	123	137	150	NA

A denotes use of anesthesia during collection of auditory evoked potentials.

- denotes a frequency not tested

^a^ Only one value for L4 (mean of L4 and L4 A) was used for this calculation

### Aerial audiogram

**Fig 5:**
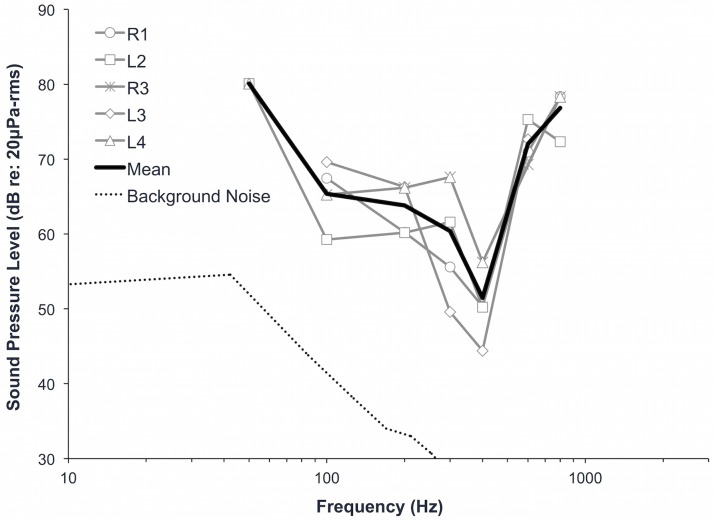
Aerial audiograms for juvenile green sea turtles (*Chelonia mydas*). Spectrum level background noise is represented the dotted line (dB re: 20 μPa/√Hz).

Juvenile green turtles responded to signals between 50 and 800 Hz in air, with maximum sensitivity at 400 Hz (Table 2, Fig 5); sensitivity decreased sharply above 400 Hz. The lowest pressure sensitivity recorded was 44 dB re: 20 μPa-rms at 400 Hz (turtle L3). We found variation among individual turtles in terms of threshold levels as well as lowest and highest frequency detected. We found variation among individuals ranging from <1 to 18 dB re: 20 μPa in air, however up to 6 dB of this variability could be due to the step size used in the AEP measurements. Four turtles responded to 800 Hz, and two turtles responded to 50 Hz. Background noise levels were <50 dB re: 20 μPa at 50 Hz, <28 dB re: 20 μPa at 300 Hz, and decreased with increasing frequency.

**Table 2:** Aerial pressure thresholds (dB re: 20 μPa-rms) for individual juvenile green sea turtles (*Chelonia mydas*), and mean thresholds for all turtles combined. Frequencies tested with no detected auditory evoked potential response are presented with > “highest sound pressure level presented” (dB re: 1 μPa-rms).

Turtle	Frequency (Hz)								
ID	50	100	200	300	400	600	800	1600	3200
R1	>76	67	60	56	50	71	78	>61	>53
L2	80	59	60	62	50	75	72	>60	>49
R3	>80	65	66	68	56	69	78	>60	-
L3	>72	70	66	50	44	73	>78	>63	>58
L4	80	65	66	68	56	-	78	>60	-
Mean	80	65	64	60	51	72	77	NA	NA

- denotes a frequency not tested

### Auditory evoked potentials using anesthesia

The sample size was too small to perform an inferential statistical analysis, but audiograms for the two anesthetized turtles did not differ greatly from those tested without anesthesia and we observed no differences in AEP waveform characteristics or latency periods. For the turtle (L4) tested with and without anesthesia, differences between resulting audiograms varied between 3 to 12 dB, however, as previously stated, up to 6 dB of this difference could be due to the step size used during AEP collection. Some turtles resisted manual restraint (L3), rendering the collection of AEPs impossible. For these turtles anesthesia was less stressful as a restraint method, as evidenced by their better blood oxygen and lower lactate levels (see Harms et al. [20] for details).

## Discussion

### Green sea turtle hearing sensitivity

Juvenile green sea turtles have a narrow range of low frequency hearing underwater and in air. Turtles responded to underwater signals between 50 Hz to 1600 Hz, with maximum sensitivity between 200 and 400 Hz. In air, turtles responded to a narrower range of frequencies, between 50 and 800 Hz, with maximum sensitivity between 300 and 400 Hz. Our underwater sound pressure threshold levels and frequencies of maximum sensitivity are similar to those measured by Bartol and Ketten [3] for partially submerged Pacific sub-adult green turtles, but we found an expanded hearing range (Bartol and Ketten: 100–500 Hz, current study: 50–1600 Hz). The frequencies of maximum sensitivity and hearing range underwater are not consistent with those measured by Bartol and Ketten [3] for partially submerged juvenile Atlantic green turtles. This difference may be due to variation in submergence levels (partially versus fully submerged), differences in stimulus, and/or population specific variability in hearing sensitivity. Ridgway et al. [1] measured responses to cochlear potentials and not AEPs, so it is difficult to compare threshold levels in air, but the frequencies of maximum sensitivity found using both techniques were similar. Unlike Ridgway et al. we did not detect hearing sensitivity above 800 Hz in air, perhaps because our stimulus level was not high enough to elicit a response. The maximum aerial sound pressure level at 1600 Hz was 63 dB re: 20 μPa-rms, to which the turtles did not produce a detectable AEP.

Green sea turtle AEP waveforms exhibited a frequency-doubling response at all frequencies tested, which has also been observed in other studies of fish, invertebrates, and sea turtle AEPs [4–5, 22–24]. In fish and invertebrates it has been hypothesized that the doubling effect is a result of differing hair cell orientation on the sensory epithelium of the otolith sac in the inner ear, causing some hair cells to fire during the compression phase of a sound wave and others to fire on the rarefaction phase, resulting in a doubled response. Sea turtle inner ears have cochleae, rather than otoliths, but a differing orientation of limbic and basilar membrane hair cells, as found in freshwater turtles (*Chysemys scripta elegans*: [9]), may cause a similar double firing and doubled response in turtles.

It is challenging to evaluate responses to low-frequency stimuli with AEP techniques. Peak background and electrical noise levels occur at very low frequencies (<200 Hz), so it can be difficult to differentiate low-frequency peaks in the FFT caused by AEP presence from those caused by background noise. Our determined thresholds at low frequencies are likely conservative. Background noise in this study likely masked low-frequency stimuli, also resulting in higher thresholds for low frequencies where background noise was <20 dB lower than threshold levels. Critical ratios have not been examined in turtles. Given the prevalence of low-frequency natural and anthropogenic sound in marine and terrestrial environments, we believe that future investigations of masking and critical ratios would be extremely useful in determining the potential impacts of these low-frequency sounds on turtle hearing sensitivity.

### Anesthesia technique and effects of anesthesia

Concurrently with this study, we developed a safe and effective technique to anesthetize sea turtles underwater to allow the collection of underwater AEPs without myogenic artifact. The technique is described in detail in Harms et al. [20], and includes measurement of venous pH, blood gases, and lactate before and after AEPs in both anesthetized and manually restrained turtles. Anesthesia was helpful to eliminate myogenic artifact in turtles that were not amenable to manual restraint (i.e., turtles that did not rest quietly throughout the procedure, moving only to raise the head to breathe), but chemical restraint was not required for all turtles. Manual restraint was superior to anesthesia for turtles that did not resist restraint due to better venous blood oxygenation and acceptable AEPs without the possibility of drug effects, but anesthesia was superior to manual restraint for turtles that resisted restraint, which exhibited marked lactic acidosis and for which AEP collection was not possible [20]. We found small differences (<12 dB re: 1 uPa, Fig 4) at several frequencies in the audiograms for the turtle for which we implemented both chemical and manual restraint techniques, but we cannot determine whether these differences were potentially due to the anesthetic or the presence of the endotracheal tube’s inflatable cuffs. We recommend further research to determine if anesthesia has a significant effect on the measured hearing sensitivity of sea turtles, as this technique may be required to collect AEPs in other juvenile and adult sea turtles.

### Comparison of underwater and aerial hearing sensitivities

**Fig 6:**
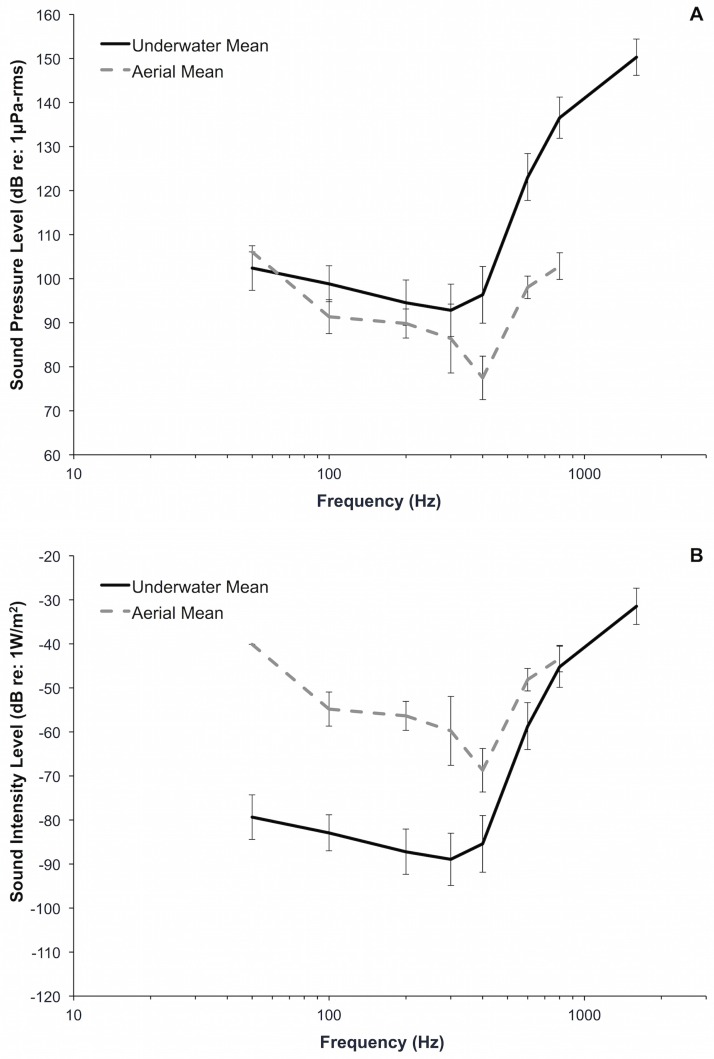
Comparison of mean (± 1 SD) underwater and aerial audiograms for juvenile green sea turtles (*Chelonia mydas*) in terms of pressure (A) and intensity (B).

The overall patterns of underwater and aerial audiograms of juvenile green sea turtles were similar, but the range of sensitivity and frequencies of maximum sensitivity were different. When thresholds were adjusted for reference pressures, green sea turtles exhibited lower sound pressure level thresholds in air, particularly at higher frequencies (Fig 6A). Below 400 Hz, aerial and underwater sound pressure level thresholds were quite similar. Aerial mean sound pressure level thresholds were lower (range: 5–34 dB) for all frequencies except for 50 Hz, where the mean aerial sound pressure level threshold was 4 dB higher than the underwater sound pressure threshold level.

To understand whether turtles may have better hearing sensitivity in air versus water, we examined hearing in terms of the amount of energy needed by a far-field source to generate a detectable pressure signal. For this analysis, we assume that sea turtles are sensitive to acoustic pressure, rather than acoustic intensity or particle motion. After pressure thresholds were adjusted by a total of 61 dB for the differences in reference pressures (26 dB) and acoustic impedance (35 dB) between the two media, sound intensity level thresholds were lower (range: 2–39 dB) underwater (less sound energy is required for detection), particularly at frequencies below 400 Hz (Fig 6B). Christensen-Dalsgaard et al. [17] also observed lower aerial threshold levels (5–12 dB) when comparing aerial and underwater hearing sensitivity relative to sound pressure and lower underwater threshold levels when comparing aerial and underwater sound intensity in the red-eared slider (*Trachemys scripta elegans*), suggesting that turtle ears respond to lower sound energy and are more efficient in water.

The presence of an air-filled middle ear suggests that pressure likely plays some role in detection of acoustic stimuli. Christensen-Dalsgaard et al. [17] hypothesized that turtles are pressure sensitive and that the turtle air-filled middle ear resonates with the aerial and underwater sound field and it is these pulsations that cause the tympanic disc (and the extracollumela and columella) to move, rather than the displacement of the tympanum directly. Lenhardt et al. [10, 13] proposed that the sea turtle ear is specialized for bone conduction, however Hetherington [14] suggested a more standard tympanic middle ear path, given that the middle and inner ears are encased in bone, restricting sound input to the oval window, further noting that marine turtles lack a heavy, inertially sensitive stapedial footplate. Christensen-Dalsgaard et al. [17] also concluded that a specialization in bone conduction is unlikely given that low particle velocities in aquatic environments would elicit small vibrations causing an ear specialized for bone conduction to respond only to high-intensity sound levels at close ranges. As a sea turtle dives, air in the lungs is pushed into the reinforced trachea, which connects to the middle ear air-filled cavity via the Eustachian tube. Bony encasing of the ear may minimize bone conduction of sound to the inner ear by restricting sound reception to the tympanum and preventing the collapse of the air-filled middle ear during deep dives, thus allowing sea turtles to hear at depth [14]. However, if the air cavity in the middle ear is compressed under pressure during deep dives and sea turtles are sensitive to only sound pressure facilitated by an air-filled middle ear, hearing sensitivity is likely to decrease dramatically at depth. It is possible that sea turtles detect and respond to both pressure and particle motion (via bone conduction or vibratory hearing, or detection by balance organs in the ear), or that one component is detected at very low frequencies and another component is detected at higher frequencies. Experiments that are able to spatially separate acoustic pressure and intensity are needed to determine the components of sound sea turtles are able detect.

### Conclusion

Juvenile green sea turtles detect low-frequency acoustic stimuli both underwater and in air. The biological significance of hearing in sea turtles remains poorly understood, but as low-frequency sound is most prevalent and travels the farthest in the marine environment there may be some advantage to sea turtles in specializing in low-frequency sound detection. As acoustic stimuli may provide important environmental cues for sea turtles, additional research is needed to determine how sea turtles behaviorally and physiologically respond to natural and anthropogenic sounds in their environment.
